# Towards AR-assisted visualisation and guidance for imaging of dental decay

**DOI:** 10.1049/htl.2019.0082

**Published:** 2019-11-26

**Authors:** Yaxuan Zhou, Paul Yoo, Yingru Feng, Aditya Sankar, Alireza Sadr, Eric J. Seibel

**Affiliations:** 1 Department of Electrical and Computer Engineering, University of Washington, Seattle, WA 98195, USA; 2 Human Photonics Lab, Department of Mechanical Engineering, University of Washington, Seattle, WA 98195, USA; 3 Paul G. Allen School of Computer Science and Engineering, University of Washington, Seattle, WA 98195, USA; 4 School of Dentistry, University of Washington, Seattle, WA 98195, USA

**Keywords:** patient treatment, enamels, medical image processing, dentistry, data visualisation, augmented reality, untreated dental decay, prevalent dental problem, significant economic burden, social burden, irreversible effects, expensive restorative treatment, early decay management difficult, unreliable detection, quantitative monitoring, early decay therapy, situ visualisation, pre-operative optically based dental images, augmented guidance, repetitive imaging, therapy monitoring, mitigate hand–eye coordination problems, help guide monitoring, tooth decay management

## Abstract

Untreated dental decay is the most prevalent dental problem in the world, affecting up to 2.4 billion people and leading to a significant economic and social burden. Early detection can greatly mitigate irreversible effects of dental decay, avoiding the need for expensive restorative treatment that forever disrupts the enamel protective layer of teeth. However, two key challenges exist that make early decay management difficult: unreliable detection and lack of quantitative monitoring during treatment. New optically based imaging through the enamel provides the dentist a safe means to detect, locate, and monitor the healing process. This work explores the use of an augmented reality (AR) headset to improve the workflow of early decay therapy and monitoring. The proposed workflow includes two novel AR-enabled features: (i) in situ visualisation of pre-operative optically based dental images and (ii) augmented guidance for repetitive imaging during therapy monitoring. The workflow is designed to minimise distraction, mitigate hand–eye coordination problems, and help guide monitoring of early decay during therapy in both clinical and mobile environments. The results from quantitative evaluations as well as a formative qualitative user study uncover the potentials of the proposed system and indicate that AR can serve as a promising tool in tooth decay management.

## 1 Introduction

**Fig. 1:**
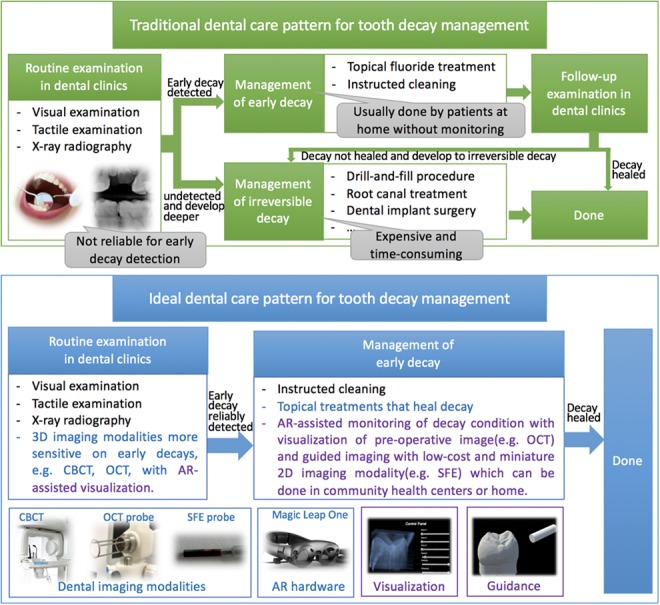
Comparison of traditional and ideal dental care patterns for tooth decay management. Blue texts are areas that are under active development. Purple texts indicate how our work is supporting the new approach to healing dental decays

Oral health problems remain a major public health challenge worldwide in the past 30 years, leading to economic and social burden [1–3]. Wherein, untreated dental decay is the most prevalent issue and is relevant to socio-economic disparities [4, 5]. As shown in Fig. 1, the traditional dental care pattern for dental decay management consists of routine examination in clinics, non-destructive treatments for detected early decays and destructive treatments for irreversible decays. There are three limitations to this pattern. First, visual or tactile examination and the current gold-standard x-ray radiography cannot reliably and timely detect interproximal and occlusal lesions [6], which are the most common types of dental decays. Second, medicine therapy and instructed cleaning are performed by patients at home without supervision. And they need to revisit the dental clinic, which limits the timely monitoring of decay and often leads to further progression of the decay into irreversible decay. Lastly, the treatments for irreversible lesion such as drill-and-fill procedure, root canal treatment and even dental implant are all destructive, painful, expensive and time-consuming. These limitations need to be solved to develop an ideal dental care procedure for decay management, also shown in Fig. 1. If early-stage lesions can be detected reliably, patients can be prescribed with medicinal therapies and instructed/directed cleaning over time outside the dental clinic [3, 7, 8]. Also, if the current clinic-revisiting-based monitoring of decay can be enhanced by monitoring at community health centre or even patient's home and sharing data with dentists, then timely intervention can be made with fewer clinic-visits and less burden on both dentists and patients [3, 9]. Then, early decays can be detected and healed in time thus avoiding destructive and costly procedures. In need is the continuous research into such ideal management of tooth decay [3].

To move towards this ideal pattern, there have been significant strides towards developing reliable, sensitive and low-cost imaging modalities to diagnose early decays [10, 11]. Three-dimensional (3D) imaging modalities such as cone-beam computed tomography (CBCT) and optical coherence tomography (OCT) are reliable and sensitive but usually require long imaging time on expensive clinical systems. Clinicians typically perform 3D imaging pre-operatively and use the 3D image for planning and intra-operative reference. For intra-operative imaging and also remote monitoring, clinicians also need a 2D imaging modality, e.g. the scanning fibre endoscope (SFE).

Along with the development of imaging modalities, the ease of use for dental imaging needs to be improved in general. Acquiring high-quality images from desired perspective usually requires expert manipulation of the instrument. For example, to effectively monitor the condition of a carious lesion with SFE, users need to image the decay from the same perspective every time, which is difficult without any assistance [12]. Also, using the previous images for navigation requires hand–eye coordination. Clinicians need to divert their attention to the display monitor while manually positioning the scope, additionally compensating for patient's movement. This is particularly challenging in dental field as there is only manual fixation of patient's jaw and patients are typically not under local anaesthesia during dental procedures. The above challenges lead to a lengthy learning curve for providing treatment accurately [13, 14]. Moreover, resource-limited areas may lack budgets for well-trained personnel.

In this work, we utilise an augmented reality (AR) head-mounted display (HMD) to develop a platform for visualising dental images from multiple modalities. We also use the HMD as a guidance tool for positioning of an imaging probe during repetitive monitoring of dental lesions and their treatments. We built a prototype system using the Magic Leap One AR headset and two dental imaging modalities OCT and infrared SFE. The key contributions of our work are (i) the design and development of a novel end-to-end system for multi-modal dental image visualisation, (ii) a technique for guided image capture using SFE, and (iii) quantitative evaluations as well as a user study to evaluate the usefulness, usability and limitations of our system and identify areas for future work.

To the best knowledge of the authors, this is the first pilot study to develop HMD-based AR environment for visualisation and guidance for optically monitoring the status of dental lesions. Continued advances in AR devices, dental imaging modalities, as well as systems that combine these two technologies will together push the traditional dental practice towards an ideal future.

## 2 Related work

**Fig. 2:**
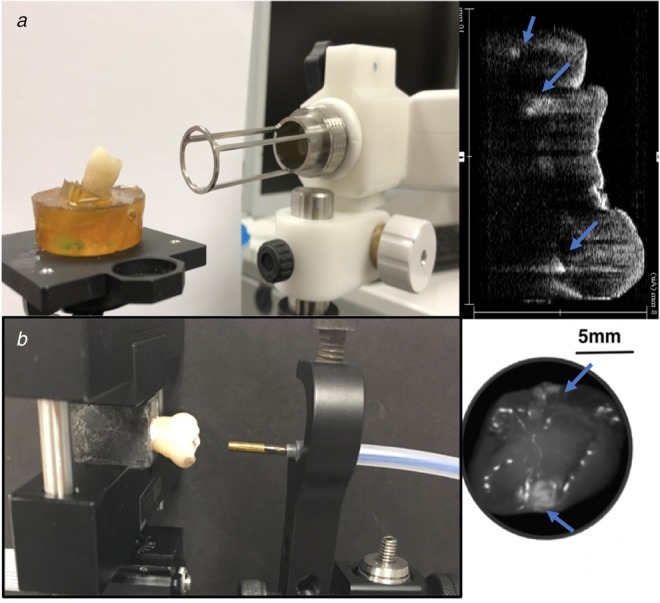
Demonstration of two NIR dental imaging modalities and their images *a* An OCT probe imaging an extracted human tooth; a slice of the 3D OCT scan, where the bright patterns indicate demineralised regions of enamel (dental lesion) *b* An SFE probe imaging an extracted human tooth; SFE image, where the bright patterns (marked by arrows) indicate high optical reflectance from dental decay regions

Near-infrared (NIR) optical imaging is shown to have the potential to detect early-stage dental decays more reliably [15, 16]. In NIR reflection image, dental decays appear brighter than surrounding sound areas due to increasing scattering coefficient [17]. OCT is a 3D volumetric imaging technique and has been used for NIR imaging of dental decay [18]. Fig. 2*a* shows a prototype OCT system imaging an extracted human tooth and a slice of the 3D OCT scan where two interproximal dental lesions appear as bright spots. OCT systems are expected to be expensive when introduced to dental clinics, and currently a complete 3D scan takes at least several minutes from prototype systems. Also, the OCT probe is bulky and requires expert manipulation to acquire high-quality scans. Thus OCT is more suitable as the pre-operative imaging modality used in clinics. The SFE is a 2D imaging technique with the advantages of miniature probe tip and expected low cost. Many SFE prototypes have been used for real-time NIR dental imaging in previous works [19–21]. Fig. 2*b* shows SFE imaging an extracted human tooth and the SFE image where the white patterns on both sides of tooth indicate two interproximal dental lesions. In the figure, SFE is imaging from the biting surface of tooth, but since NIR light penetrates around 3 mm deep into the surface [20], the interproximal dental lesion under the surface also shows up in the image. This is very helpful for dental decays that are hidden in between the neighbouring teeth and not accessible to the operator. Due to the above advantages, SFE is well-suited for quick intraoperative screening and long-term monitoring.

AR technology has been introduced into research areas of dental implant [22–26], oral and maxillofacial surgery [14, 27–29], orthodontics [30] as well as dental education [31, 32]. In previous work, introduction of AR has assisted clinicians by displaying and registering virtual models in the operating field thus reducing difficulty of hand–eye coordination. However, there is as yet no study aimed at assisting dental imaging modalities for detection and monitoring of dental decay [33]. Among all available AR devices, HMDs have the advantage of compactness and intuitiveness (as compared to handheld or armature mounted AR devices). For this study, we chose Magic Leap One [34] AR headset as the hardware platform. Magic Leap One also includes a hand-held controller with a home button, a bumper, a trigger and a touchpad.

## 3 Methods

**Fig. 3:**
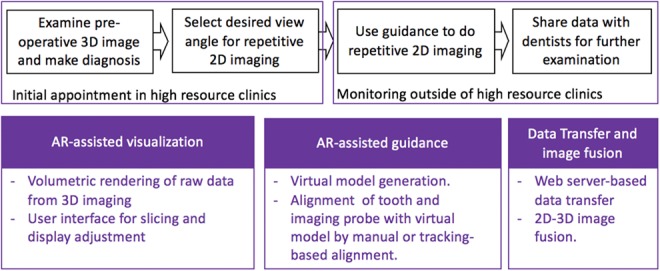
Diagram of workflow and corresponding technical components

The proposed workflow and corresponding technical components are described in Fig. 3. During the initial appointment in dental clinics with high resource availability, a pre-operative 3D raw image is acquired and transferred onto AR headset, and then dentists can examine the 3D image in AR environment intraoperatively and make a diagnosis based on observed position, dimension and severity of dental decays. During this process, the dentist can translate, rotate, and scale the 3D image at will to view it from an optimal viewing angle based on their preference and experience. The dentist can also adjust display parameters including intensity, opacity, and contrast threshold to optimise decay visibility and also account for varying external lighting conditions. Furthermore, they can examine the image by slicing through the 3D structure to accurately locate the decay.

For long-term monitoring, the dentist can select the desired angle of view for future repetitive 2D imaging. Then a virtual model of tooth and imaging instrument, with registered spatial relationships, is generated and stored. During the monitoring phase, 2D imaging can be performed regularly within or outside of a clinical setting, using the virtual model as guidance. In order to reproduce the reference image, the operator aligns the position of the selected tooth and the imaging probe with respect to the virtual model so that the same desired view angle is preserved. Alignment of imaging probe can be done by manual alignment or tracking-based alignment. 2D images are then transferred into AR environment and fused with the 3D image and all previous 2D images for comparison. The operator or remote dentist can change the desired angle of view according to updated 2D images throughout the period of monitoring. After 2D SFE images are acquired, they are fused with 3D image and transferred to a dentist with computer-aided image analysis for interpretation. By comparing the historical images to the present, the dentist can make determination of whether the dental decay is healing or is progressing under the current prescription and make corresponding adjustment on the prescription (such as frequency and dose of medicine application, and/or time of next dental visit). We prototyped a software system based on this principle using Unity [35] (version 2019.1.0f1) with Magic Leap Lumin SDK [34].

### 3.1 AR-assisted visualisation of pre-operative 3D image

In our pilot study, a pre-operative 3D image of the tooth is acquired using a pre-commercial 1310 nm swept-source OCT (Yoshida Dental Mfg., Tokyo, Japan) with 110 nm band and 50kHz scan. The OCT 3D scan is taken from the occlusal view with an imaging range of $10 \times 10 \times 8\, {\rm m}{\rm m}^3$ and an axial imaging resolution of 11 µm. The raw data from OCT imaging system is first converted into point cloud data and downsampled to reduce the data size without losing useful features. The point intensities are then rescaled to increase the dynamic range. The point cloud data is then rendered as a 3D volumetric object using an open-source Unity package for volumetric rendering [36].

Slicing through three orthogonal directions is implemented to allow users to inspect inner structures of the tooth. By examining cross-section slices, dentists can comprehensively inspect the location and size of dental lesions. More importantly, dentists can find out how deep the dental decay has progressed into the dental enamel layer, which would determine whether a drill-and-fill procedure is needed or the medicine treatment should be prescribed with long-term monitoring. Since the visualisation needs to accommodate different lighting conditions and user preferences, adjustment of three display parameters is provided. Users can adjust intensity value to adjust the overall brightness of the volumetric display. They can also adjust the threshold value for saturation, hiding areas that have low contrast. Opacity value can be adjusted to determine the transparency of the volume. Appropriate opacity values allow the user to see the surface structure of tooth as well as inner features like dental decay or a crack without having to inspect through every slice, thus providing an initial and intuitive sense of existence, position and structure of these features. Slicing and display adjustment are implemented as sliders on a panel. The controller is used to select and adjust sliders. The panel and the pre-operative 3D image can be selected by aiming the controller at them and holding down the trigger and physically translating or rotating the controller. When the panel or the image is selected, users can also rescale them by pressing on left of the touchpad to shrink and left of the touchpad to enlarge. See the video in supplementary material for the interaction demo.

### 3.2 AR-assisted guidance for 2D imaging

**Fig. 4:**
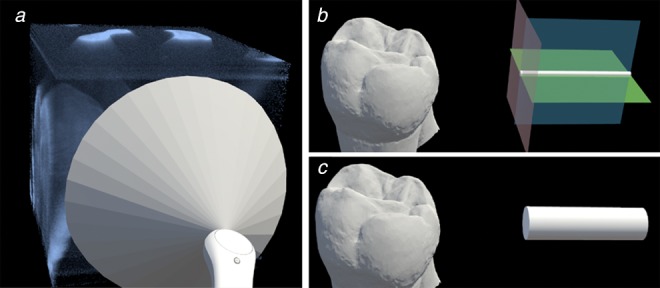
Design of view selection and probe models *a* Use cone model to select desired angular view for consistent 2D imaging *b* The tri-colour-plane model for probe alignment *c* The cylinder model for probe alignment

Guidance for 2D imaging is necessary not only in that it helps non-dentist personnel to take 2D images at desired view angles, but also in that it guarantees the field of view and perspective of 2D images during repetitive imaging remain the constant and the series of images can be quantitatively compared. After dentists spot decay on the OCT 3D image, they can designate the desired view angle to take 2D images so that the decay can be detected by 2D images. In the view angle selection mode, a virtual cone shape is attached to the end of controller, corresponding to the view frustum of the endoscope. Since NIR SFE has a disc-shaped field of view which grows larger when the target is further away from the probe, a cone can be used to represent the field of view of SFE. The user can aim the cone at the OCT 3D image and adjust the area that is covered by the cone, as shown in Fig. 4*a*. By pressing the bumper to indicate that the desired view angle is chosen and a virtual reference model consisting of 3D tooth surface model registered with SFE probe model according to indicated view angle is generated for future guidance. The 3D tooth surface model is acquired by an intra-oral scanner (3Shape TRIOS 3, 3Shape, Copenhagen, Denmark).

In this pilot study, we strive to keep the system and workflow as concise as possible, so we are not using any fiducial-point-based tracking which requires an additional tracker. Furthermore, the alignment between the virtual tooth model with the real tooth is done manually by the user. Since the virtual tooth model is the 3D surface structure scan from the same tooth, the user can shrink the model to the same size as the tooth and align them. The next step is to use the reference model for guidance of 2D imaging, where the user needs to align the virtual probe model. The alignment of SFE probe to the virtual model is made more difficult since SFE probe is of a smaller scale. Therefore, we designed two virtual SFE probe models, a cylinder model and a tri-colour-plane model, as shown in Figs. 4*b* and *c*.

Besides manual alignment, there are also two tracking-based methods supported by hardware systems on Magic Leap One. The first method is based on image-tracking API provided by Magic Leap [37]. The front-view camera and depth camera on the headset can be used for tracking the spatial position and rotation of a flat image. The target image is printed in the dimension of $3.4 \times 3.2\, {\rm c}{\rm m}^2$ and attached to the SFE probe. Then the tracked position and rotation of the target image can be transformed to the position and rotation of the probe, assuming the offset between the probe and target image remains rigid and unchanged. The second method is based on the electromagnetic 6-DoF spatial tracking of the control handle [38]. By fixing the SFE probe with the control handle, the tracked position and rotation of the controller can be transformed into the position and rotation of the probe. Once the probe is being tracked, a red cylinder virtual model is shown to indicate the tracked position and rotation. Then the user needs to align the red cylinder virtual model (the tracked position and rotation of the real probe) with the virtual probe model (desired position and rotation for positioning the real probe).

### 3.3 Data transfer and image fusion

**Fig. 5:**
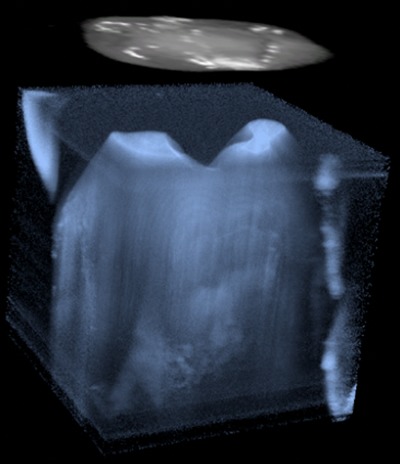
Fusion of OCT 3D image and SFE 2D images

The 2D SFE images are transferred from the instrument to the AR headset via a web server. A polling-based scheme downloads newly acquired images onto the headset, over HTTP. 2D SFE images and the 3D OCT image can then be registered according to the view angles with which the SFE images were taken. As shown in Fig. 5, an occlusal-view SFE image is registered with the OCT 3D image. With the image fusion, users can interpret and compare images from multiple modalities and also inspect the condition of decays during monitoring of therapy.

## 4 Evaluation

### 4.1 Experiments

**Fig. 6:**
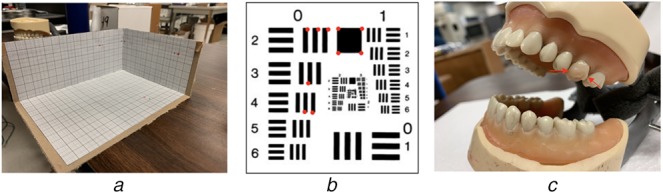
Experiment setup *a* 3D grid coordinate for measuring augmentation accuracy between hologram and object *b* USAF resolution test chart for measuring end-to-end accuracy during probe repositioning. Ten key points are selected from square corners marked by red dots *c* Dentoform model with an extracted human tooth installed on top. There are two artificial dental decays on the interproximal surfaces marked by the two red arrows

To measure the augmentation quality, we set up a 3D grid coordinate as shown in Fig. 6*a*. The grid paper has 1 mm fine grids, 5 mm medium grids and 1 cm large grids. Once the hologram is manually aligned with the object, the observer uses a sharp pointer to localise position of a certain point on hologram and then measures the distance between the points on real object and hologram. Jitter and perceived drift of the hologram are quantified by the translation distance measured on the grid paper.

To measure the alignment performance, we also measure the end-to-end accuracy quantified by keypoint displacement in acquired SFE images. We choose to image a USAF resolution test chart as shown in Fig. 6*b*, to simplify the accurate extraction of keypoints in SFE images. Ten key points are selected on the test chart. The user first aligns the SFE probe in front of the test chart in desired viewpoint and takes one image. Then after putting the SFE probe down for a while, the user realigns the SFE probe with or without guidance and takes another SFE image with the attempt to replicate the same viewpoint as in the first image. Three guidance approaches are used in turn for the guidance of repositioning of SFE probe, among which, ‘without any guidance’ means that user aligns the probe only according to their memory of the desired probe position without referring to real-time SFE video, ‘with AR guidance’ means that user aligns the probe with the AR hint of desired probe position, ‘with video guidance’ means that user aligns the probe by referring to the real-time SFE video and comparing with the reference image. Three guidance approaches are used in random order for ten runs to avoid training bias. The time it takes to realign the probe to desired position is recorded. The *x* and *y* positions of the *i*th keypoint are measured in pixels in reference image and repetitive image as $p_{x^i}^{{\rm ref}} $, $p_{y^i}^{{\rm ref}} $, $p_{x^i}^{{\rm rep}} $, $p_{y^i}^{{\rm rep}} $. The overall keypoint displacement *D* of the repetitive image is then calculated according to

$$D = \displaystyle{{\sum\nolimits_i \sqrt {{\lpar p_{x^i}^{{\rm rep}} - p_{x^i}^{{\rm ref}} \rpar }^2 + {\lpar p_{y^i}^{{\rm rep}} - p_{y^i}^{{\rm ref}} \rpar }^2} } \over {10}}$$

Among ten runs, the mean and standard deviation of *D* is quantified and used to evaluate the three guidance approaches along with the time.

### 4.2 User study

**Fig. 7:**
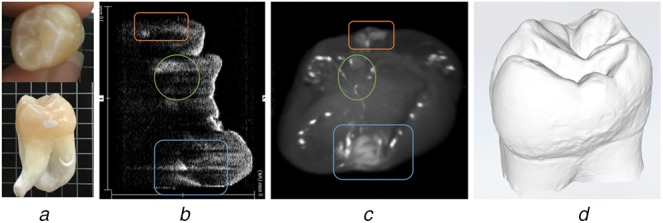
Extracted human tooth with artificial interproximal decays *a* Photograph of the extracted human tooth with two artificial interproximal lesions *b* One slice of OCT 3D image of the tooth *c* NIR occlusal-view SFE image *d* 3D surface shape scan of the tooth. Note that in (*b*) and (*c*), the blue frame indicates an artificial dental decay deep into the dentin, the orange frame indicates an artificial dental decay less than halfway into the enamel, and the green circle indicates a natural dental decay in the groove under the biting surface

We conducted a user study to get user feedbacks for this prototype. We used a dentoform model with an extracted human tooth installed on it, as shown in Fig. 6*c*. The extracted human tooth has two artificial dental lesions on its interproximal surfaces. OCT 3D image, occlusal-view SFE 2D image as well as 3D surface shape scan were acquired from this sample, as shown in Fig. 7.

Six subjects were recruited and asked to conduct the tasks with the system, to walk through the workflow. Among the six subjects, three self-reported as dental students or clinicians, while the other three were general users without specialised dental knowledge. All users were new to this AR system and the workflow. The protocol that subjects were asked to perform using the Magic Leap One were as follows: (i) examine the 3D OCT image in the headset by slicing and adjusting display parameters. (ii) Use the cone to select the desired view angle. (iii) Manually align the virtual model with the real tooth. (iv) Align the SFE probe with the virtual probe model and compare two virtual probe models. The manual alignment, image-tracking-based alignment and controller-tracking-based alignment are also compared.

After the tasks were completed, the users were asked to fill out a questionnaire anonymously. See supplementary material for the template of questionnaire.

## 5 Results and discussion

In the quantitative measurements, we measured the augmentation quality between hologram and objects manually aligned together. We noticed the augmentation quality is influenced by jitter, perceived drift and latency, which degrade perception as well as accuracy and efficiency of the alignment procedure. Jitter is the continuous shaking of the hologram. We measured jitter within the range of 1 mm, which is at the edge of our acceptable range considering the tooth to have a dimension of around 10 mm. Perceived drift is that when the observer moves around a hologram, the perceived position of hologram drifts away. We measured the perceived drift within the range of 5 mm when the observer takes two orthogonal viewpoints. The perceived drift limits users from observing from multiple viewpoints to align probe with the hologram. However, considering that users are not able to freely move around when aligning the probe, the perceived drift may be less fatal for our prototype. Latency is the time lag of hologram update when the user moves their head and is determined by the distance of head movement. The measured latency is within range of 2 s when head motion is within the general range needed for performing the imaging procedure. We also measured the accuracy of image-tracking-based alignment and controller-tracking-based alignment. The image-tracking-based alignment suffers from limited capability of front-facing camera. The image tracking has an error of up to 4 mm and may lose the target when the printed target image moves fast. Furthermore, when the background of environment is complicated, the image tracking may recognise the wrong target. It is recommended that the image tracking is used in well-lit space while avoiding black or very uniform surfaces as well as reflective surface like mirrors or glasses. The controller-tracking-based alignment suffers from the hologram drift when the electromagnetic sensor is rotated around or moved close to conducting surfaces. All that being said, the current image-tracking and controller-tracking-based alignment approaches suffer from instability and accuracy issues and need improvement either from hardware or from the tracking scheme design. So far, manual alignment seems to be more robust in terms of accuracy and efficiency.

The end-to-end accuracy and efficiency of manual alignment is quantified by the keypoint displacement in acquired reference SFE image and repetitive SFE image with dimension of 400 × 400 pixels. As shown in Table 1, AR guidance has the advantage of better repositioning accuracy compared to without any guidance, and the advantage of faster repositioning speed compared to using SFE real-time video for guidance. By transferring the real-time SFE video to AR headset and placing it near the operating field, we may further improve the accuracy and efficiency of our prototype.

**Table 1:** Comparison of different imaging guidance approaches

Imaging guidance approach	keypoint displacement, px	Time taken, s
without any guidance	83 ± 10	3
with AR guidance	31 ± 11	10
with video guidance	7 ± 2	20

In the user study, the average time taken to educate each subject to use the system to general proficiency (i.e. familiar with the interaction techniques and can use them to accomplish the workflow) was 15 min, which is quite fast considering their unfamiliarity to AR devices. Afterwards, all subjects were able to accomplish the protocol. During the process of prototyping and quantitative evaluation, we thought the following factors may influence the workflow and therefore included qualitative questions regarding their effects. The factors include (i) the latency which may impede the accuracy and efficiency of alignment of the tooth and probe with the virtual models due to the small scale, (ii) the available field of view of the headset. For Magic Leap One, the width and height of the AR field of view are currently the largest in the market and the interface design also avoid borders of frames to mitigate the sense of the limited field of view. However, when the user is too close to the virtual objects, the virtual objects will be cut off by a clipping plane. This limits users to work from a distance of about 37 cm away from the virtual objects, which means that the users may have to always extend their arms away from their body during the alignment tasks. Five subjects felt the latency was noticeable but it did not impede their workflow, while one dental clinician felt the latency of the headset was an impediment. Five subjects reported that the limits of the AR field of view within the headset were unnoticeable, while only one general user thought clipping plane of the headset caused discomfort/distraction during the workflow.

As for feedback on the workflow, three dental personnel all thought the AR-assisted visualisation of OCT is an improvement over standard screen display in the sense of flexible movement in space while preserving the same information as the standard display. Two dental clinicians that are familiar with the OCT image were able to localise the position of both artificial interproximal lesions (decay) and even the natural decay in the groove. The other dental student is not familiar with OCT images so was not able to do this. Although, they commented that the rendering speed of OCT image may be a problem when more 3D scans need to be acquired. All three dental personnel and one general user thought the SFE 2D imaging AR-assisted guidance is easier than without guidance, while two other general users thought it was more difficult. These two general users commented that the manual alignment of the virtual tooth model and the real tooth is complicated due to one major reason. The depth perception does not work well when you want to accurately align virtual object with real object. This is caused by an inherent issue called occlusion leak which has also been reported for other AR devices like Hololens [39] and there's ongoing research on solving this issue [40]. The image tracking and controller tracking sometimes also suffer from instability. The choice of manual alignment versus tracking-based alignment methods seems to be up to personal preference. In terms of choice of virtual probe model, all three general users prefer the tri-colour-plane model, while three dental personnel have various preference. Therefore it is advantageous to have both virtual probe models available and provide an interface to switch between the two.

This first-ever prototype showed both clinical potential and technical limitations in our study, which we believe will be a useful reference for future research. First, the AR display can relieve clinicians or general users from the troubles of constantly switching views between patient and computer screen and the consequent hand–eye coordination problem. Importantly, the AR display preserves required information in the composite images. Second, this system can assist in the adaptation of multiple dental imaging modalities into clinical use, such as the safe and informative infrared optical imaging. Since images from multiple modalities can be integrated into the system and provide supplementary information for clinicians, this improves the learning curve of clinicians on using these new imaging modalities, and also improves the reliability and sensitivity of dental decay quantification. Notably, the prototype can be easily generalised to other dental imaging modalities available in the clinics, such as CBCT, NIR and fluorescence dental cameras. Also, most of these imaging modalities along with the intra-oral scanners are common in dental clinics. The SFE we use in this study is not commercial but expected to be a low-cost NIR imaging modality. The other addition is the AR headset which continues to get cheaper. Thus, our prototype is both generalisable and cost-effective. Lastly, the proposed solution can help repetitive imaging of dental decay for therapy monitoring, which is the core of the ideal dental care protocol of tooth decay management which maintains the integrity of teeth. There are definite limitations in our prototype reported above. Some limitations stem from the inherent restrictions of the Magic Leap One hardware, such as jitter, perceived drift, latency, occlusion leak and limited FOV. We believe that the rapid progress of AR HMD products will help resolve these limitations. Other limitations stem from our designs on the software and workflow themselves, such as the inaccuracy of manual alignment, which may be resolved by improved designs of tracking mechanism. See supplementary material for the video demo of our system in use.

## 6 Conclusion

In this work, we proposed an AR-assisted visualisation and guidance system for imaging of dental decay. We introduce a novel workflow which is implemented as a software application on the Magic Leap One AR headset. We evaluated the multimodal system and workflow through quantitative measurements as well as a pilot user study with the recognition that the prototype can be generalisable to other more conventional dental imaging modalities, such as 3D-CBCT and 2D-oral cameras. Thus, with the addition of an AR headset and a low-cost 2D imaging modality like SFE, our prototype can be adapted into dental clinics and rural community health centres.

## 7 Funding and declaration of interests

Financial support was provided by US NSF PFI:BIC 1631146 award and VerAvanti Inc. Equipment support was provided by NIH/NIDCR R21DE025356 grant and Yoshida Dental Mfg. Corp. A.S. was supported by the University of Washington (UW) Reality Lab, Facebook, Google, and Huawei. Authors have no personal conflicts of interest outside the UW. UW receives license and funding from Magic Leap Inc., and VerAvanti has licensed SFE patents from UW for medical.
